# Diffuse terahertz spectroscopy in turbid media using a wavelet-based bimodality spectral analysis

**DOI:** 10.1038/s41598-021-02068-7

**Published:** 2021-11-23

**Authors:** Mahmoud E. Khani, Omar B. Osman, M. Hassan Arbab

**Affiliations:** Department of Biomedical Engineering, Stony Brook University, 100 Nicolls Rd, Stony Brook, NY 11794 USA

**Keywords:** Terahertz optics, Applied optics, Biophotonics, Imaging and sensing, Optical spectroscopy

## Abstract

Current terahertz (THz) spectroscopy techniques only use the coherent light beam for spectral imaging. In the presence of electromagnetic scattering, however, the scattering-mitigated incoherent beams allow for flexible emitter-detector geometries, which enable applications such as seeing through turbid media. Despite this potential, THz spectroscopy using diffuse waves has not been demonstrated. The main obstacles are the very poor signal to noise ratios of the diffused fields and the resonance-like spectral artifacts due to multiple Mie scattering events that obscure the material absorption signatures. In this work, we demonstrate diffuse THz spectroscopy of a heterogeneous sample through turbid media using a novel technique based on the wavelet multiresolution analysis and the bimodality coefficient spectrum, which we define here for the first time using the skewness and kurtosis of the spectral images. The proposed method yields broadband and simultaneous material characterization at detection angles as high as 90° with respect to the incident beam. We determined the accuracy of the wavelet-based diffuse spectroscopy at oblique detection angles, by evaluating the area under the receiver operating characteristic curves, to be higher than 95%. This technique is agnostic to any a priori information on the spectral signatures of the sample materials or the characteristics of the scattering medium, and can be expanded for other broadband spectroscopic modalities.

## Introduction

Diffuse optical imaging techniques have made it possible to investigate objects hidden beneath opaque scattering layers^1,2^. The spatial^3^ and temporal^4^ control of light propagation through an inhomogeneous turbid medium using wavefront-shaping techniques^5^ have enabled super-resolution imaging in applications such as fluorescence microscopy^6^. Computational techniques based on the analysis of the speckle-pattern correlation functions have simplified the experimental geometries needed for single-shot narrowband imaging through scattering layers^7^. Additionally, computational methods such as diffusing photon tomography^8^, diffusing temporal-field correlation analysis^9,10^, and speckle-pattern tomography through correlations of the light’s interference patterns^11^ have enabled narrowband diffuse spectroscopy for investigating the heterogeneity in dynamic turbid media. However, these diffuse optical spectroscopy techniques have been limited to narrowband systems and therefore lacked broadband spectroscopic capabilities for material characterization. In contrast, the temporal correlations of the diffused broadband THz electric fields have been utilized to characterize single scattering events in a turbid medium^12,13^. Yet, broadband diffuse THz spectroscopy has not been demonstrated, in part due to the spectral artifacts caused by the multiple scattering events, which can obscure the characteristic absorption resonances of the sample materials. In this work, we demonstrate diffuse THz time-domain spectroscopy (THz-TDS) at oblique detection angles using a novel computational approach based on the wavelet multiresolution analysis (MRA) of the diffuse THz extinction spectra to simultaneously resolve the resonant frequencies of different materials in a heterogeneous sample buried beneath a turbid scattering medium.

Radiation at THz frequencies can penetrate nonconducting materials that are opaque at optical wavelengths and provide sub-millimeter lateral and sub-picosecond axial resolutions^14,15^. THz light has been employed for applications ranging from noninvasive diagnosis of the skin carcinoma^16,17^ to the detection of illicit drugs and explosives^18–23^. These applications often involve seeing through turbid media^24–26^, where the presence of discrete scatterers in the form of wavelength- and sub-wavelength-size granular particles or air voids results in classical electromagnetic scattering^27^. In the electromagnetic scattering theory, traveling a distance longer than the Boltzmann transport mean free path randomizes the propagating beam into ballistic and diffusive fields^28^. THz-TDS techniques mostly use the ballistic and quasi-ballistic portion of the transmitted THz light^15^. They either ignore the diffused THz waves^29,30^ or employ them only by averaging with the ballistic forward-scattered fields^31^. For example, the effect of volume scattering in pressed pellets of materials were studied using forward-scattered THz electric-field transmission measurements^29,30,32–36^. However, utilization of only diffusely-scattered waves critical in studying hostile and uncooperative samples, or in detection geometries without access to the narrow cone of the forward-scattered beams, has not been investigated. The main obstacles in diffuse THz spectroscopy are the very poor signal to noise ratios (SNR) of the scattered fields^31,37^ and the appearance of scattering-induced artifacts in the form of wavelength-dependent extinction and anomalous spectral features.

Here, we demonstrate for the first time diffuse spectroscopy using the spectrally encoded oblique THz beams with high sensitivity and specificity. We present a robust computational approach to identify the constituent chemicals of a heterogeneous sample buried beneath a turbid scattering medium. Our experimental methodology consisted of reconfiguration of a THz-TDS transmission setup to collect both the ballistic and diffused rays by scanning a 180$^{\circ }$-half-plane field-of-view. We employed the wavelet multiresolution analysis to extract the spectral signatures from the diffuse extinction spectra, which presented a complicated superposition of the materials’ characteristic responses and the scattering-induced artifacts. The extinction spectra were reconstructed from wavelet bases at specific scales, which were selected by minimizing a cost function defined using the total variation in the bimodality coefficients of the spectral images. We discovered that the bimodality coefficient spectrum, defined using the skewness and kurtosis of the spectral images, leads to simultaneous broadband recognition of the absorption resonant frequencies. We demonstrate the accuracy of our method through the component spatial pattern analysis^18,38,39^, achieving an area under the Receiver Operating Characteristic (ROC) curve better than 0.99 in the forward-scattered beams, and 0.95 using only the diffuse THz beams at oblique angles.

## Results

### Diffuse THz spectroscopy

**Figure 1:**
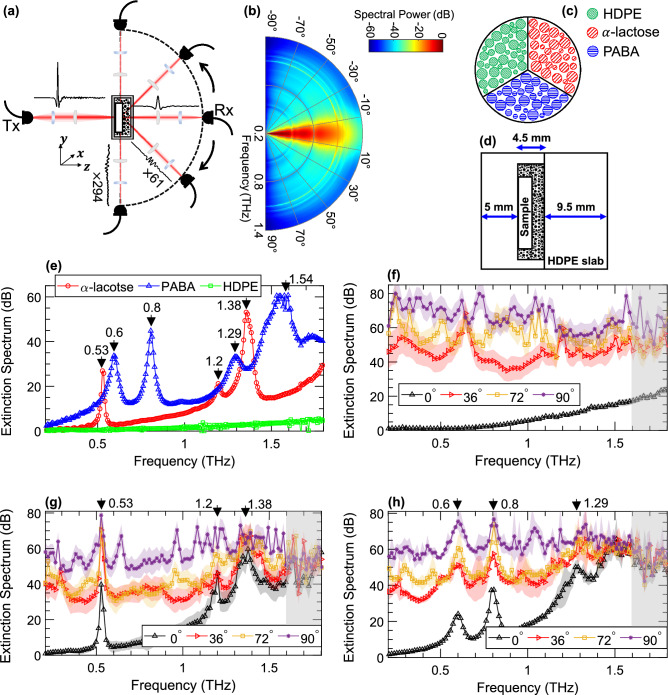
Diffuse-wave THz-TDS, (**a**) a schematic of the imaging apparatus designed for the measurement of the ballistic and diffuse THz fields, example THz-TDS signals, multiplied by the normalization factors for visualization purposes only, are given at different angles, (**b**) the angular distribution of the scattered THz spectral power (jet color map generated in MATLAB R2020b, https://www.mathworks.com/products/matlab) measured through the sample embedded in the loosely-packed LDPE powders, showing a 60 dB decline from $\theta = \text {0}^\circ$ to 90°, (**c**) a schematic presentation of the imaging target outlining the regions covered by each chemical (i.e., HDPE (green circles), $\alpha$-lactose (red circles), and PABA (blue circles)), (**d**) a schematic of the sample housing made from two HDPE slabs, a reservoir is made into one of the slabs to contain the sample pellet and the turbid medium surrounding it, (**e**) the extinction spectrum of the chemicals in the imaging target, measured without any scattering layers in the beam path, revealing the characteristic resonant frequencies, (**f**-**h**) the extinction spectra measured at four different detection angles including $\theta = \text {0}^\circ$, 36°, 72°, and 90° from the pixels containing HDPE (the green circles in (**c**)), $\alpha$-lactose monohydrate (the red circles in (**c**)), and PABA (blue circles in (**c**)), respectively, each line in (**f**-**h**) shows the average extinction coefficient along its standard deviation over the pixels associated with each chemical.

Our experimental apparatus consisted of a modified THz spectrometer, shown in Fig. 1a, for obtaining THz-TDS measurements using both ballistic and diffuse THz waves. In the emission arm, THz pulses generated by optical excitation of a photoconductive antenna (PCA) using 1560 nm pulses of a femtosecond laser were collimated and focused on the imaging target. As it is shown in the schematic of the measurement setup in Fig. 1a, the detection arm composed of the collimating and focusing lenses and the fiber-coupled PCA detector was rotated in a 180$^{\circ }$ half-plane around the center axis of the sample cell and recorded signals at a step size of $\Delta \theta =18^{\circ }$. This step size was selected based on the beam divergence angle, which was calculated using the diameter of the collimated beam and the focal length of the focusing lens, to ensure no overlap between measurements at adjacent angles^31,40^. Figure 1a also illustrates an example incident THz pulse along with waveforms recorded at forward and oblique detection angles. It can be noticed that the diffused time-domain pulses do not resemble a typical single-cycle THz pulse. They instead exhibit a superposition of multiple-scattered and attenuated fields with different times of arrival^13^. For visualization, signals at oblique angles were multiplied by normalization factors, given in Fig. 1a for two representative detection angles. Figure 1b illustrates the angular distribution of the THz spectral power measured through the imaging target. It can be noted that the power of the scattered radiation is confined significantly in the narrow cone of the forward-scattered waves, and the SNR declines by 60 dB from $\theta =0^{\circ }$ to $\theta =90^{\circ }$. In the supplementary note 1, we discuss an analytical formalism for the prediction of angular scattering patterns using the Mie scattering theory. In this work, we present a novel computational approach for extraction of reliable spectroscopic information from diffuse beams at arbitrary detection angles as high as 90$^{\circ }$.

The imaging target was a sample disk with its area divided into three regions, as shown in Fig. 1c, where each region was filled with a different chemical, namely $\alpha$-lactose monohydrate, 4-aminobenzoic acid (PABA), and high-density polyethylene (HDPE), covering the red, blue and green regions in Fig. 1c, respectively. Additionally, the sample disk was embedded inside a turbid scattering medium, composed of loosely-packed low-density polyethylene (LDPE) powders with 180 $\upmu$m mean particle size, yielding a 0.3 g/cm$^3$ volume density. The target was contained in a sample cell made from two 9.5 mm-thick HDPE slabs, where a 4.5 mm-thick reservoir was machined into one of the slabs, as shown in Fig. 1d. Therefore, the average thickness of the scattering layer over the sample disk was 2.5 mm. Figure 1e compares the extinction spectra of the sample component chemicals, where the distinct resonant signatures of $\alpha$-lactose and PABA can be observed having different spectral shapes and properties. These shapes of the absorption features are characterized using a resonant signature’s height and its full width at half maximum (FWHM) in the Lorentz oscillator model^41^. Unlike $\alpha$-lactose and PABA, polyethylene (HDPE or LDPE) is transparent at THz frequencies and does not have any characteristic resonances. The extinction coefficients in Fig. 1e are measured using separate sample pellets made of each chemical (i.e., $\alpha$-lactose monohydrate, PABA, and HDPE). These measurements are acquired in transmission geometry without any scattering layers in the beam path. The transmission measurements through the samples are deconvolved by a reference measurement through the air to calculate the extinction coefficients. To form spectroscopic images the sample housing containing the imaging target and the scattering medium was raster scanned at each detection angle, resulting in $30\times 30$-pixel spatiotemporal THz data with pixel size of $2\times 2$ mm$^2$, corresponding to the beam size at the focus.

The approximations to the solution of the Maxwell’s equations applied to the problem of electromagnetic scattering by spherical particles are divided into three domains based on the dimensionless size parameter $x=2\pi nr/\lambda$, where $2\pi r$ is the circumference of the scattering particle, and $\lambda /n$ is the wavelength of the incident radiation in the medium. Based on the value of *x*, the relevant scattering regimes are referred to as the Rayleigh scattering ($x\ll 1$), Mie scattering ($x\approx 1$), and geometric scattering ($x\gg 1$). Here, we find that $x = 0.38-7.54$ when $r = 180$ $\mu$m (for the LDPE scattering layer) in the frequency range between $f = 0.1-2$ THz ($\lambda = 3{\text{ mm}}{-}150\;{\upmu }{\text{m}}$). This range covers more than an order of magnitude variation in the size parameter due to the broadband nature of the THz radiation in a THz-TDS system. In contrast, the average size of the HDPE particles in the pellet sample, as reported by the manufacturer, was 4.5 $\upmu$m, while the median size of the $\alpha$-lactose monohydrate and PABA powders was 8 $\upmu$m and 7 $\upmu$m, respectively. Therefore, electromagnetic scattering by HDPE, $\alpha$-lactose monohydrate, and PABA powders lies on the boundary of Rayleigh and Mie scattering regimes. For a more detailed discussion of the scattering regimes, we refer the reader to the Supplementary Information 1.

Figure 1f-h exhibit the diffuse spectroscopy of the sample component chemicals through the extinction spectra measured at different detection angles. They compare the extinction spectra of HDPE, $\alpha$-lactose monohydrate, and PABA, respectively, at $\theta =0^{\circ }, 36^{\circ }, 72^{\circ }, \text {and } 90^{\circ }$. Each line in Fig. 1f-h represents the average extinction spectrum of each material over its corresponding pixels, along with its standard deviation. It can be noted that in addition to a 60 dB drop in the signals’ spectral power from $\theta =0^\circ$ to $\theta =90^\circ$, the characteristic absorption signatures of the $\alpha$-lactose monohydrate and PABA were obscured by the scattering-induced spectral artifacts at diffuse angles. Moreover, averaging over multiple pixels associated with each material, which is considered a standard technique for eliminating the scattering artifacts^33^, has not improved the oblique-angle measurements. In Fig. 1g-h, the observation of resonances at higher frequencies is even further hindered due to the frequency-dependent nature of the multiple scattering effects. These spectral artifacts are further discussed in the supplementary note 1. Results in Fig. 1g-h confirm that in the oblique-angle measurement geometries the extinction spectra represent a superposition of a material’s characteristic response and the scattering-induced deterministic Mie resonances, which will impede accurate spectroscopic analysis.

**Figure 2:**
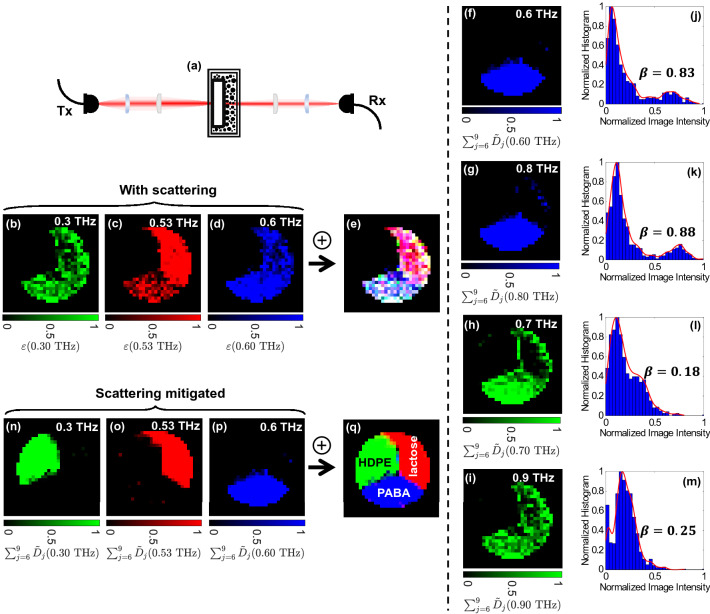
Terahertz chemical mapping through turbid media, (**a**) the imaging apparatus at the detection angle $\theta =0^{\circ }$, (**b**) the normalized extinction coefficient at 0.3 THz (green color map), (**c**) 0.53 THz (red color map), (**d**) 0.6 THz (blue color map), (**e**) the superposition of the images in (**b**-**d**), (**f**) image formed using the normalized summation of the sixth to ninth-level wavelet details vectors at 0.6 THz (i.e., $\sum _{j=6}^9\tilde{D}_j$(0.6 THz)), (**g**) $\sum _{j=6}^9\tilde{D}_j$(0.8 THz), (**h**) $\sum _{j=6}^9\tilde{D}_j$(0.7 THz), (**i**) $\sum _{j=6}^9\tilde{D}_j$(0.9 THz), (**j**-**m**) the normalized histograms of the images shown in (**f**-**i**), respectively, the bimodality coefficient value is given for each histogram, (**n**) the normalized summation of the sixth to ninth-level wavelet details vectors at 0.3 THz (green color map), (**o**) 0.53 THz (red color map), (p) 0.6 THz (blue color map), and (**q**) the superposition of the images in (**n**-**p**). The color maps are generated using MATLAB R2020b, https://www.mathworks.com/products/matlab.

Figure 2 illustrates the challenges in spectroscopic imaging through a turbid scattering medium, even at forward-scattered geometries, as shown schematically in Fig. 2a. Figure 2b-d exhibit the images of the normalized extinction coefficients formed at 0.3 THz (no absorption lines—control experiment), 0.53 THz (lactose’s resonant mode), and 0.6 THz (PABA’s resonant mode) at detection angle $\theta =0^{\circ }$ in the presence of the scattering layer. It can be observed that the area covered by each chemical is not clearly discernible in these images, and the image shown in Fig. 2e, which shows the superposition of the images in Fig. 2b-d, does not provide an accurate image of the sample chemicals. Moreover, as it will be shown in the following sections, the scattering effects will be more severe in diffuse spectroscopic imaging, $\theta \ne 0^{\circ }$. This is because, as shown in Fig. 1b, the diffusely scattered spectral power drops by approximately 60 dB when comparing the measurements at detection angles $\theta =0^{\circ }$ and $\theta =90^{\circ }$. Therefore, developing computational techniques to identify and remove such scattering artifacts is crucial for imaging through turbid scattering layers.

### Scattering mitigation through wavelets and bimodality spectral analysis

The electromagnetic scattering in THz-TDS measurements through inhomogeneous media can alter or obscure the characteristic spectral signatures of the sample. Various works have contributed to understanding the scattering effects on the THz spectra. In particular, it has been shown that the granular scattering in pressed pellets appears as a strong frequency- and particle size-dependent attenuation superimposed on the coherent THz fields^29^. Analytical models, such as the dense medium theory within the quasicrystalline approximation (QCA)^29^, and numerical methods incorporating the Mie partial scattering amplitudes have been developed to estimate the multiple scattering loss in granular composite materials^35^. The extinction spectra of loosely-packed nonabsorptive particles have also been modeled using a frequency-dependent power law, $\varepsilon (\nu )=\beta \nu ^A$^33,42^. Yet estimation of the scattering-induced extinction does not describe a method for removal of these effects and identification of resonant frequencies from the distorted or obscured spectral signatures. Therefore, estimating the scattering contribution in the extinction coefficient of materials with characteristic absorption signatures requires more advanced computational approaches. Moreover, previous studies have mainly focused on estimating the scattering loss in the ballistic regime^29,30,32–36^, and the effects of volume scattering on diffuse THz fields have not been investigated. Here, we present a robust computational approach based on the wavelet multiresolution analysis (MRA) of the THz extinction spectra along with the kurtosis and skewness of the spectral images, presented as a new spectroscopy concept dubbed the bimodality spectrum, to reliably identify the characteristic resonant frequencies using the scattered THz waves in both ballistic and diffusive regimes.

Wavelet transforms produce multi-resolution signal presentations by filtering a signal with a set of pre-defined wavelet and scaling filters at different scales, while preserving the locations of the distinct signal features^43–45^. Using the wavelet MRA, a discrete THz extinction spectrum, $\varepsilon (f)$, can be expressed as a linear combination of scaling and wavelet basis functions^46^. In this way, the scaling function provides a coarse-scale approximation of $\varepsilon (f)$, while the wavelet functions return its details at successive dyadic scales. The *j*th-level wavelet detail coefficients are given by,

1 $$\begin{aligned} \tilde{D}(j,f) = \sum _{k}d_{j,k}\Psi _{j,k}(f), \end{aligned}$$

where $\Psi _{j}$ represents a wavelet basis function at the normalized scale $\tau _j=2^{j-1}$, and $d_{j}$ are the wavelet coefficients generated using $\Psi _{j}$^43,44^. Here, we used the maximal overlap discrete wavelet transform (MODWT) pyramid algorithm to compute the wavelet and detail coefficients. Spectroscopic material identification using the MODWT decomposition of the THz extinction spectra is reduced to finding the wavelet bases’ scales that correctly capture the characteristic spectral features, which itself depends on the properties of a resonant signature, i.e., its height and FWHM. Here, we developed an optimization procedure based on the bimodality coefficient in the THz spectral images to find these wavelet bases objectively.

In broadband spectral imaging of a heterogeneous sample, images formed at fingerprint absorption bands yield a higher contrast^21^. The modal characteristics of an image histogram distribution can be used to assess its contrast^47^, where a multimodal distribution can indicate a higher contrast. To illustrate this idea, Fig. 2j-m compare the histogram of the images formed at the PABA resonant frequencies at 0.6 and 0.8 THz, shown in Fig. 2f,g, with the histogram of the images formed at two off-resonant frequencies, namely 0.7 and 0.9 THz, shown in Fig. 2h,i. It can be seen that the histograms at 0.6 and 0.8 THz, shown in Fig. 2j,k, demonstrate two well-separated modes, whereas the histograms at 0.7 and 0.9 THz, shown in Fig. 2l,m, are either unimodal or the two modes are very close together and overlap. To quantitatively evaluate this modal behaviour, we used the bimodality coefficient, which for an image with *n* pixels is given by^48,49^

2 $$\begin{aligned} \beta (f)=\frac{\gamma ^2(f)+1}{\kappa (f)+\frac{3(n-1)^{2}}{(n-2)(n-3)}}, \end{aligned}$$

where $\gamma (f)$ and $\kappa (f)$ represent the skewness and kurtosis of the spectral image at frequency *f*. The skewness and kurtosis are the third and fourth-order standardized moments of an image around its mean, respectively. Their corresponding equations are given in the Methods section. The skewness depends on the asymmetry of a histogram distribution, whereas kurtosis depends on its tailedness^50,51^. The value of the bimodality coefficient $\beta$ varies between 0 and 1, where a $\beta$ greater than 5/9, i.e. that of a uniform distribution, indicates either a bimodal or a multimodal histogram^52^. The bimodality coefficients of the bimodal histograms shown in Fig. 2j,k, corresponding to images formed at 0.6 and 0.8 THz resonant frequencies, are 0.83 and 0.88, respectively, whereas for the unimodal histograms shown in Fig. 2l,m, for images formed at off-resonant frequencies, the bimodality coefficient is 0.18 and 0.25. Noteworthy here, the bimodality coefficient is a function of the frequency at which each image is formed. Henceforth, we introduce the bimodality coefficient spectrum of each spectral image, which is independently calculated for measurements at different detection angles.

In order to identify the appropriate wavelet bases’ scales using the bimodality coefficient, it should be noted that many spectral images formed by including only the fine-scale wavelet detail vectors in the reconstruction of the extinction spectra will have a bimodality greater than 5/9, which results in erroneous identification of the resonant modes. However, because the fine-scale wavelet bases mostly capture the high-frequency noise and scattering artifacts in the spectrum^53^, the bimodality spectrum, i.e. $\beta (f)$ in the range $f=0.2-1.6$ THz, would be highly fluctuating with a large total variation. On the other hand, in images formed by including only the coarse-scale wavelet detail vectors in the image reconstruction, the bimodality spectrum would be smooth and slowly-varying. To balance the trade-off effect of fine-scale and coarse-scale wavelet basis functions, we implemented an optimization procedure given by,

3 $$\begin{aligned} \min _{\tilde{D}_{1},...,\tilde{D}_{J}} \quad \frac{\sum _{i}|\beta (f_{i})-\beta (f_{i-1})|}{K\sum _{i}\beta (f_{i}),\;\forall \;\beta (f_{i})>\frac{5}{9}} \end{aligned}$$

for all possible combinations of wavelet detail vectors in the MODWT MRA over *J* dyadic scales, $\tau =2^{0},2^{1},...,2^{J-1}$. The total number of such combinations is $2^J$. The numerator in Eq. (3) represents the total variation of the $\beta (f)$ along the spectrum. The denominator accounts for the bimodalities greater than 5/9, and *K* represents the number of included levels of wavelet details. We find that after reconstruction of the extinction spectra by including only the wavelet details that minimize Eq. (3), the local maxima greater than 5/9 in the bimodality spectrum reveal all the signature resonant frequencies of the imaging target’s constituent materials. Importantly, it should be noted that the wavelet-based reconstruction of the image and its bimodality spectrum does not benefit from any a priori information on the type and nature of the chemicals in the sample.

**Figure 3:**
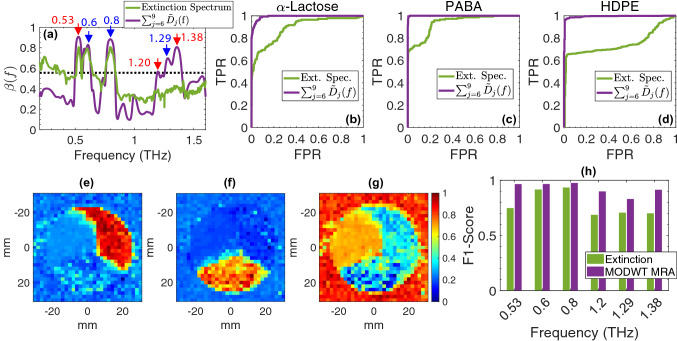
The bimodality-based MODWT MRA at $\theta =0^{\circ }$, (**a**) the bimodality coefficient of the images formed from the extinction spectra and the MODWT MRA in the range 0.2–1.6 THz, (**b**-**d**) the receiver operating characteristic (ROC) curves obtained for identification of the region of each chemical using the component spatial pattern analysis based on the extinction spectra and the bimodality-based MODWT MRA, (**e**-**g**) the chemical maps (jet color map generated in MATLAB R2020b, https://www.mathworks.com/products/matlab) obtained using the component spatial pattern analysis based on the MODWT details for $\alpha$-lactose, PABA, and HDPE, respectively, (**h**) comparison of the F1-scores obtained for chemical mapping at each resonant frequency using the extinction spectra and the MODWT detail vectors.

Figure 2n-p show the normalized summation of the sixth to ninth-level wavelet details vectors at 0.3 THz ($\sum _{j=6}^9\tilde{D}_j$(0.3 THz)), 0.53 THz ($\sum _{j=6}^9\tilde{D}_j$(0.53 THz)), and 0.6 THz ($\sum _{j=6}^9\tilde{D}_j$(0.6 THz)) . It can be observed that, unlike Fig. 2b-e, these images yield the exact regions covered by each chemical. Moreover, as it is shown in Fig. 3a and is further discussed in Performance evaluation section, rather than identifying the characteristic resonant modes using the extinction spectrum at each pixel separately, we achieve chemical recognition by examining the local maxima in the bimodality spectrum. In other words, the bimodality spectrum can simultaneously distinguish between multiple resonant signatures, even those in close spectral proximity, such as the $\alpha$-lactose’s resonance at 0.53 THz and the PABA’s resonance at 0.6 THz. In the supplementary note 2, we investigate the robustness of this technique to change in the spectral shape of the absorption features and the proximity of two adjacent resonant modes. Finally, it should be noted that this technique does not rely on spatial averaging, unlike previous work^33,37,54^, to remove the scattering effects. This is a critical advantage for our approach because spatial averaging over disjointed measurements does not lend itself to imaging applications and is not suitable for characterization of heterogeneous samples made from multiple chemicals, where the area covered by each material is not known in advance.

### Performance evaluation

#### Ballistic scattering

Solving Eq. (3) for the extinction spectra measured in the $30\times 30$ pixels at $\theta _0=0^{\circ }$ reveals that reconstruction of $\varepsilon (f)$ from only the sixth to ninth-level MODWT details, i.e. $\tilde{D}_{6}(f)+\tilde{D}_{7}(f)+\tilde{D}_{8}(f)+\tilde{D}_{9}(f)$, results in simultaneous recognition of all the resonant frequencies of $\alpha$-lactose and PABA in the bimodality spectrum. Figure 3a compares the bimodality coefficients of the images formed from the original extinction spectra with those of the images formed from the reconstructed images using the specific combination of MODWT details. The red arrows in Fig. 3a point to the $\alpha$-lactose’s resonances at 0.53, 1.20, and 1.38 THz, while the blue arrows indicate the PABA’s resonances at 0.6, 0.8, and 1.29 THz. It can be noticed that in the bimodality spectrum of the MODWT details (purple line), it is only the local maxima corresponding to the resonant frequencies of the sample materials that exhibit $\beta$ greater than the 5/9 threshold.

However, in the bimodality spectrum of the extinction spectra (green line), although values at 0.53, 0.6, and 0.8 THz are greater than 5/9, the higher-frequency resonances at 1.20, 1.29, and 1.38 THz are completely obscured due to scattering effects. Moreover, the bimodality spectrum of the MODWT details remains below the 5/9 threshold at all other frequencies except the exact resonant frequencies of the samples, whereas the bimodality spectrum formed by the extinction spectra is still greater than 5/9 at obfuscate frequencies between 0.2 and 0.4 THz.

Furthermore, to quantitatively validate the effectiveness of this method in removing the scattering effects in spectroscopic imaging, we used the component spatial pattern analysis (CSPA) approach, which is described in the Methods section, to identify the region covered by each chemical^18,38,39^. Figure 3b-d compare the receiver operating characteristic (ROC) curves, demonstrating the true positive rate (TPR) versus the false positive rate (FPR), achieved in classifying the pixels associated with each chemical using the CSPA approach based on the extinction spectra and the bimodality-based MODWT MRA. Derivation of these ROC curves is described in the Methods section. It can be noted that the MODWT MRA yields area under the curve (AUC) better than 99$\%$, whereas using the raw extinction spectra can only give AUC values below 80$\%$ due to the scattering artifacts. In other words, the bimodality and wavelet-based reconstruction of the spectral images improved the accuracy of material mapping to about almost 100$\%$. Finally, it should be noted that the CSPA technique rather than utilizing the extinction at a specific frequency, e.g., a resonant frequency, uses the entire spectrum at each pixel, i.e., $\varepsilon (f)$ for $f=0.2-1.6$ THz, to calculate the probability that a pixel belongs to each of the component chemicals. Figure 3e-g illustrate such chemical maps obtained for each of the materials in the imaging target, i.e., $\alpha$-lactose monohydrate, PABA, and HDPE, respectively, using the MODWT detail vectors. Here, the color code between 0 and 1 represents the probability that a pixel belongs to any of the three chemicals in Fig. 3e-g. Figure 3h compares the F1-scores, the harmonic mean of sensitivity and specificity, in identifying the region of each chemical using the extinction spectra and MODWT details at the resonant frequencies. It can be observed that the F1-scores achieved using the MODWT details are significantly higher than those obtained using the extinction spectra, especially at 0.53, 0.6, and 0.8 THz, where the F1-scores are all above 96%. Moreover, the F1-scores at 1.2, 1.29, and 1.38 THz are each improved by at least 20%, reaching to 91% at 1.2 and 1.38 THz from below 70%. These results verify the effectiveness of the bimodality-based wavelet MRA technique for THz spectroscopy and spectral imaging applications using the ballistic waves measured at the narrow-cone of the forward angle.

#### Diffuse scattering

**Figure 4:**
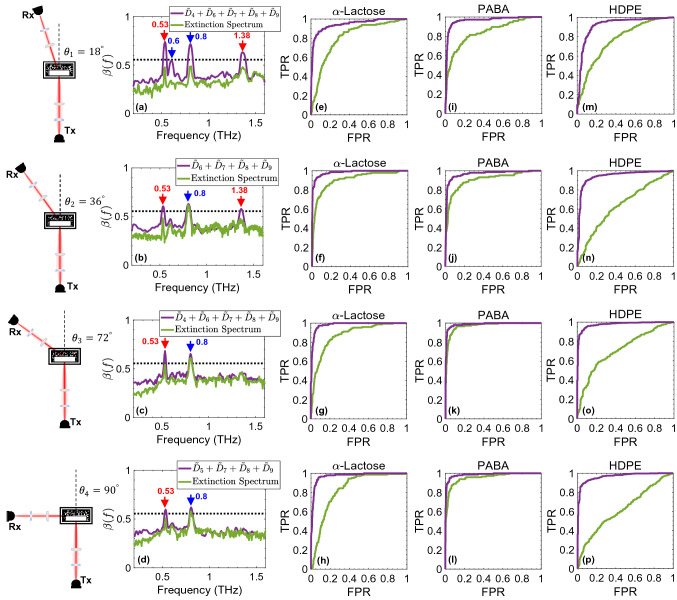
Diffuse THz spectroscopy, (**a**-**d**) the bimodality spectra obtained using the diffuse extinction spectra and their MODWT detail vectors at the oblique detection angles including $\theta _1=18^{\circ }$, $\theta _2=36^{\circ }$, $\theta _3=72^{\circ }$, and $\theta _4=90^{\circ }$, (**e**-**h**) the ROC curves obtained for the identification of the region of $\alpha$-lactose using the diffuse extinction spectra and their MODWT detail vectors at each oblique detection angle, (**i**-**l**) similar to (**e**-**h**) for the identification of the region of PABA, (**m**-**p**) similar to (**e**-**h**) for the identification of the region of HDPE.

As we have described in the experimental design, the detection angles were chosen carefully to avoid any overlaps between the measurements taken at adjacent angles. Therefore, signals measured at oblique angles were only associated with the diffuse scattering of the THz waves. We also showed that because of the multiple scattering artifacts and the very low SNR of the diffuse waves, identifying the characteristic resonances in diffuse spectra was hindered. Here, we investigate the effectiveness of the bimodality spectrum and bimodality-based wavelet reconstruction in diffuse THz spectroscopy and imaging at higher detection angles. Figure 4 illustrates the results at four oblique detection geometries, including $\theta _{1}=18^{\circ }$, $\theta _{2}=36^{\circ }$, $\theta _{3}=72^{\circ }$, and $\theta _{4}=90^{\circ }$, which are all within the incoherent diffuse scattering regime, as demonstrated in the supplementary note 1.

Figure 4a-d show that a specific combination of MODWT detail vectors exists at each detection angle, as the solution to the optimization criteria in Eq. (3), such that in the resultant bimodality spectrum, the thresholded local maxima reveal the characteristic resonances resolved by the radiation scattered to that angle. Figure 4a-d compare such bimodality coefficients formed using the specific combination of MODWT details with those of the raw diffuse extinction spectra. The specific combinations of MODWT details, which are given in the legends of Fig. 4a-d, are determined by minimizing Eq. (3) over the measurements taken at $30\times 30$ pixels of each detection geometry.

In Fig. 4a, i.e., the detection angle $\theta _{1} = 18^{\circ }$, in the bimodality spectrum of the MODWT detail vectors, the local maxima above the 5/9 threshold correspond to the resonant frequencies at 0.53, 0.6, 0.8, and 1.38 THz. By increasing the detection angle to $\theta _{2} = 36^{\circ }$ in Fig. 4b, the bimodality spectrum at 0.6 THz falls below the 5/9 threshold. However, it can be noticed that the resonant frequency at 1.38 THz is still distinguishable as a thresholded local maximum. By further increasing the detection angle to $\theta _{3} = 72^{\circ }$ and $\theta _{4} = 90^{\circ }$, the bimodality spectrum still surpasses the 5/9 threshold at lower-frequency resonances at 0.53 and 0.8 THz. In other words, using the bimodality spectrum and the wavelet MRA approach, both materials, i.e., $\alpha$-lactose monohydrate and PABA, could still be identified, even from the radiation scattered to $\theta _{4}=90^{\circ }$. It also can be observed that in the measurements at $\theta _{3} = 72^{\circ }$ and $\theta _{4} = 90^{\circ }$, resonant frequencies above 0.8 THz could not be resolved. This can be explained by the angular scattering patterns described in supplementary note 1, where we have shown that the radiation at lower frequencies is scattered to higher detection angles more significantly, while the angular cross-section of the higher-frequency radiation is narrower. Therefore, the higher-frequency resonances were not detected in the measurements at $\theta _{3} = 72^{\circ }$ and $\theta _{4} = 90^{\circ }$. Finally, it should be noted that the PABA’s resonance at 0.6 THz was only identified at $\theta _{1} = 36^{\circ }$, whereas the 0.8 THz-resonance was identified in all oblique detection geometries. This can be explained by the proximity of the $\alpha$-lactose’s resonance at 0.53 THz and the PABA’s resonance at 0.6 THz, and indicates that in addition to the shape of a resonant signature, bimodality-based detection approach is also affected by the proximity of adjacent resonances in a sample. In the supplementary note 2, we further discuss this effect and investigate the sensitivity of this approach to the location of adjacent resonant signatures.

To evaluate the improvements in diffuse spectroscopic imaging, Fig. 4 compares the ROC curves derived from the CSPA approach using the extinction spectra, shown by the green lines, and the MODWT detail vectors, shown by the purple lines. The ROC curves demonstrate the accuracy of classifying $\alpha$-lactose in Fig. 4e-h, PABA in Fig. 4i-l, and HDPE in Fig. 4m-p, in respective columns and for each detection angle in each row. It can be noticed that the area under the ROC curves is significantly higher for the three materials when using the unique MODWT detail vectors combination instead of the raw extinction spectra, yielding classification accuracy values always higher than 90%. These results confirm the robustness of the bimodality-based wavelet MRA approach for material detection and chemical mapping using diffuse THz spectroscopy, despite the 60 dB decline in the SNR at higher detection angles.

## Discussion

Despite the significant promise of powerful spectroscopic techniques in the THz band, many real-world applications have not been realized yet. One of the key limiting factors is the effect of volume and Mie scattering on the transmission spectra. These effects result in obscured or distorted resonant signatures and the appearance of anomalous spectral artifacts. In this work, we demonstrated, for the first time, broadband diffuse THz spectroscopy using radiation incoherently-scattered by the sub-wavelength-size particles in a turbid medium. We presented the implementation of a wavelet multiresolution analysis technique to identify the component chemicals in a mixture of heterogeneous substances with different dielectric functions. We introduced a bimodality-based wavelet reconstruction approach for removing the scattering effects in the diffuse THz extinction spectra. The bimodality coefficient spectrum, which was defined using the skewness and kurtosis of the spectral images, was also utilized for simultaneous recognition of multiple resonant frequencies. We demonstrated for the first time that the local maxima above a certain threshold in the bimodality spectrum are associated with the fingerprint resonant frequencies. Additionally, minimization of the total variation in the bimodality spectrum over included levels of wavelet details in the MRA reconstruction yielded the unique wavelet bases whose combination mitigated the scattering-induced spectral artifacts.

We showed the effectiveness of this approach in resolving resonant frequencies at detection angles as high as 90$^\circ$ with respect to the incident beam, where the measurements showed a 60 dB decline in the SNR compared to the ballistic and forward-scattered signals measured at $\theta _0=0^\circ$. Importantly, by alleviating the need for any spatial or angular averaging of many measurements to mitigate the scattering effects, this technique reduces the acquisition time and is suitable for real-time broadband spectral imaging applications. Moreover, this approach can achieve scattering mitigation and material identification simultaneously without relying on any a priori information. One key limitation of our technique lies in its requirement for the component materials of the sample to be heterogeneous with distinct dielectric functions such that the bimodality coefficient spectrum, which is a measure of the image contrast, can be accurately calculated. Finally, as shown through simulations in Supplemental Figs. 6 to 8 in the Supplementary Information, the identification of multiple materials is a more challenging task. The effectiveness of this technique in identification of multiple resonances depends on the shape of the resonant signatures and their spectral proximity. However, while our technique is not limited by the number of sample materials with THz spectral fingerprints, the robustness of this approach depends upon the shape and amount of spectral overlap between the dielectric resonances of the target materials.

## Methods

### Experimental design

#### Measurement setup

We used a commercial fiber-laser THz time-domain spectrometer, TeraSmart (Menlo Systems, Inc., Newton, NJ, USA), for acquiring the THz-TDS measurements. TeraSmart uses the 1560 nm pulses of a femtosecond laser at a 100 MHz repetition rate and a 70 mW output power, split into two beams used for the synchronized generation and detection of the THz waveforms. In the emission arm, THz pulses, generated using the emitter photoconductive antenna (PCA), were first collimated using a TPX50 lens (Menlo Systems, Inc.) with 50 mm focal length, and were then focused on the imaging target using a PTFE100 lens (Thorlabs, Inc., Newton, NJ, USA) with 100 mm focal length. The size of the Gaussian beam at the focus was approximately 2 mm using knife-edge measurements. In the detection arm, two lenses identical to those in the emission arm were used for the collimation and refocusing of the transmitted or scattered THz waves on the detector PCA. The PTFE lenses with larger focal length enabled the rotation of the detection arm in a $180^\circ$ half-plane around the sample housing. At each detection angle, the sample housing was raster-scanned using two orthogonal linear translation stages (Zaber Technologies, Vancouver, BC, Canada).

#### Imaging target

The imaging target, a mixture sample disk with 50 mm diameter, was composed of $\alpha$-lactose monohydrate (Spectrum Chemical Mfg, Gardena, CA, USA), 4-aminobenzoic acid (PABA) (Sigma-Aldrich Corp., St. Louis, MO, USA), and high-density polyethylene (HDPE) (Mirco Powders Inc., Tarrytown, NY, USA). To make the sample disk, we divided the area of a pellet pressing die into three regions, and each region was filled with a different substance. The HDPE region contained 2 g of ultra-fine HDPE with 4.5 $\upmu$m mean particle size. The two other regions contained 1 gr of each chemical, i.e., $\alpha$-lactose monohydrate or PABA, diluted in 1 gr of HDPE, which was added as a binding agent. The mixture of the three regions was pressed under 3000 psi load for 30 min. The pressed disk was embedded inside 5 gr of loosely-packed low-density polyethylene (LDPE) powders with 180 $\upmu$m mean particle size as the turbid scattering medium. The sample disk and the scattering medium were contained inside a housing made from two 9.5 mm-thick HDPE slabs, where a square-shaped powder cell with 22.5 cm$^{3}$ volume was machined into one of the slabs. The filling factor of the scattering medium, which is defined as the ratio of the particles to the empty cell volume, was approximately 0.3. The average thickness of the scattering medium on top of the sample in the powder cell was 2.5 mm. In order to measure the extinction coefficients in Fig. 1e, separate pellets of $\alpha$-lactose monohydrate and PABA were made by mixing 2 g of each chemical with 2 g of HDPE, which was added as the binding agent. The mixture was pressed under 3000 psi pressure for approximately 30 min, yielding pellets with 25 mm radius and approximately 2 mm thickness.

### Wavelet multiresolution analysis

In wavelet multiresolution analysis (MRA), a discrete broadband extinction spectrum, $\varepsilon (f)$, which is evaluated at *N* frequency points, can be expressed as the linear combination of a scaling function, $\Phi _{J}$, and wavelet basis functions at various scales, $\Psi _{j}$ where $j=1, \ldots , J$. This linear combination is given by^43,55^,

4 $$\begin{aligned} \varepsilon (f) = \sum _{k}C_{J,k}\Phi _{J,k}(f)+\sum _{j=1}^ {J}\sum _{k}d_{j,k}\Psi _{j,k}(f), \end{aligned}$$

where *j* and *k* are the scale and translation parameters, respectively. The $C_{J}$ are the *J*th level approximation (scaling) coefficients of $\varepsilon (f)$. These coefficients are associated with the local averages of $\varepsilon (f)$ over the scale $\tau _{J}=\sigma .2^{J}$, where $\sigma$ is the sampling period of $\varepsilon (f)$. Moreover, the $d_{j}$ represent the *j*th level wavelet coefficients of $\varepsilon (f)$. These coefficients are associated with the differences of the weighted averages of $\varepsilon (f)$ over the scale $\tau _{j}=\sigma .2^{j-1}$. In the discrete wavelet transform (DWT), as the scale parameter changes dyadically, i.e., $\tau _j=\sigma .2^j$ for $j=1, \ldots ,J$, the translation parameter *k* also varies dyadically (down-sampled by $2^j$ at level *j*) in order to achieve a compact signal representation. However, the down-sampling operation induces error in the alignment of the wavelet-domain features with the original spectrum^45,56^. Here, we used the maximal overlap discrete wavelet transform (MODWT)-based MRA, in which the translation parameter is uniform across all decomposition levels^44,57^, ensuring exact alignment between the extracted features and the original signal^45,56^. For MODWT, Eq. (4) can be written as,

5 $$\begin{aligned} \varepsilon (f)=\sum _{k=0}^{N_{J}-1}\tilde{g}_{J}(k)\tilde{v}(J,k+f\text { mod }N)+ \sum _{j=1}^{J}\sum _{k=0}^{N_{j}-1}\tilde{h}_{j}(k)\tilde{w}(j,k+f\text { mod }N), \end{aligned}$$

where $N_J$ and $N_j$ represent the sizes of the scaling filter $\tilde{g}_{J}$ and wavelet filter $\tilde{h}_{j}$, respectively. Here, $\tilde{g}_{J}$ and $\tilde{h}_{j}$ are obtained using the inverse discrete Fourier transform (iDFT) of the transfer functions given by^44^,

6 $$\begin{aligned} \tilde{G}_{J}(m)=\prod _{l=0}^{J-1}\tilde{G}(2^{l}m), \end{aligned}$$

and

7 $$\begin{aligned} \tilde{H}_{j}(m)=\tilde{H}(2^{j-1}m)\prod _{l=0}^{J-2}\tilde{G}(2^{l}m), \end{aligned}$$

where $\tilde{G}(m)$ and $\tilde{H}(m)$ represent the transfer functions of the MODWT scaling and wavelet filters, $\tilde{g}(k)$ and $\tilde{h}(k)$ derived from a mother wavelet filter, e.g., least asymmetric (symlet) or Daubechies (db) mother wavelets^58^, as functions of the normalized frequency *m*. The ‘mod’ notation in Eq. (5) refers to the ‘modula’ operator, implying circular convolution at the boundaries. The wavelet and scaling coefficients, $\tilde{w}_j$ and $\tilde{v}_j$, in Eq. (5) were calculated using the MODWT pyramid algorithm given by^44^,

8 $$\begin{aligned} \tilde{w}(j,f)=\sum _{k=0}\tilde{h}(k)\tilde{v}(j-1,f-2^{j-1}k\text { mod } N), \end{aligned}$$

and

9 $$\begin{aligned} \tilde{v}(j,f)=\sum _{k=0}\tilde{g}(k)\tilde{v}(j-1,f-2^{j-1}k\text { mod } N). \end{aligned}$$

### Skewness and kurtosis

In the probability theory, skewness represents the third standardized moment of a probability density function around its mean, and is a measure of asymmetry in the distribution^51^. For an image with *n* pixels skewness is given by,

10 $$\begin{aligned} \gamma =\frac{\frac{1}{n}\sum _{i=1}^{n}(x_i-\bar{x})^3}{[\frac{1}{n}\sum _{i=1}^{n}(x_i-\bar{x})^2]^{3/2}}, \end{aligned}$$

where $x_i$ is the pixel intensity, and $\bar{x}$ is the average image intensity.

Kurtosis also represents the fourth standardized moment of a probability density function around its mean, and is a measure of taildness in the distribution^51^. For an image with *n* pixels kurtosis is given by,

11 $$\begin{aligned} \kappa =\frac{\frac{1}{n}\sum _{i=1}^{n}(x_i-\bar{x})^4}{[\frac{1}{n}\sum _{i=1}^{n}(x_i-\bar{x})^2]^2}. \end{aligned}$$

After finding the skewness and kurtosis for all the $30\times 30$-pixel images formed using either the extinction spectra or the MODWT detail vectors along the measurement bandwidth, i.e., $f=0.2-1.6$ THz, the bimodality spectrum is computed using Eq. (3).

### Component spatial pattern analysis

By rearranging a three-dimensional THz data structure obtained by raster scanning a sample with *L* pixels, where each pixel’s extinction spectrum is measured at *N* frequency points, into a matrix $I_{N\times L}$, the linear transformation resulting in *I* is given by^18,38,39^,

12 $$\begin{aligned} {[}I]=[S][P], \end{aligned}$$

where the columns of $S_{N\times M}$ contain the known extinction spectrum of each of the *M* chemicals at *N* frequencies. Consequently, $P_{M\times L}$ is a chemical map in which the $P_{ij}$ element represents the contribution from chemical *i* in the spectrum measured at pixel *j*. Therefore, using the extinction spectra measured at each angle in [*I*] and the known extinction spectra of the chemicals in [*S*], [*P*] is given by,

13 $$\begin{aligned}{}[P]=([S]^t[S])^{-1}[S]^t[I]. \end{aligned}$$

By feeding *P* to a sigmoid function, $\frac{1}{1+e^{-p}}$, and applying threshold values in the interval [0,1] to its outputs, two values are obtained at each threshold: the true positive rate (TPR), also known as sensitivity, and the false positive rate (FPR), which is equal to 1—specificity. The receiver operating characteristic (ROC) curves were formed using the TPR and FPR values at each threshold. The area under the ROC curve serves as a measure of the chemical mapping performance, where a higher area under the curve implies a better classification accuracy.

For the spectral images formed at the resonant frequencies, the values obtained at each pixel were fed into a sigmoid function, and its outputs were used to find the recall and precision by comparison to the known pixel labels. We used the points with the optimal recall and precision values to calculate the F1-score given in Fig. 3h.

## Supplementary information

Supplementary Information.

## Data Availability

The datasets used and analyzed during the current study are available from the corresponding author on reasonable request.
