# Supplementary material for: Diffuse terahertz spectroscopy in turbid media using a wavelet-based bimodality spectral analysis
Source: Sci Rep. 2021 Nov 23;11:22804. doi: 10.1038/s41598-021-02068-7 (PMC8611087; doi:10.1038/s41598-021-02068-7)
Supplement: Supplementary file 1 — Supplementary Information. [file 41598_2021_2068_MOESM1_ESM.pdf]

Supplementary information: Diffuse Terahertz Spectroscopy in Turbid Media through a Wavelet-based Bimodality Spectral Analysis

Mahmoud E. Khani , Omar B. osman , and M. Hassan Arbab*

Department of Biomedical Engineering, Stony Brook University, 100 Nicolls Rd, Stony Brook, NY 11794, USA
*hassan.arbab@stonybrook.edu

1 Theoretical Scattering Models

1.1 Mie theory

The Mie scattering formalism, under the independent scattering approximation, can predict the scattering contribution in the measured extinction spectra of granular solids[1]. Accordingly, the far-field angular scattering patterns created by a spherical particle can be derived using the Mie scattering amplitude functions $S_1(\theta)$ and $S_2(\theta)$ given by[2],

$$S_1(\theta) = \sum_{n=1}^{\infty} \frac{2n+1}{n(n+1)}(a_n\pi_n + b_n\tau_n), \tag{1}$$

and

$$S_2(\theta) = \sum_{n=1}^{\infty} \frac{2n+1}{n(n+1)}(a_n\tau_n + b_n\pi_n), \tag{2}$$

representing the parallel and perpendicular polarizations of the scattered field, respectively. Here, a_n and b_n are the complex coefficients of Riccati-Bessel functions and depend on the complex refractive index ratio and the size parameter $x = 2\pi r/\lambda$. Additionally, $\pi_n = p_n^1/\sin\theta$ and $\tau_n = \mathrm{d}p_n^1/\mathrm{d}\theta$, where p_n^1 is associated with Legendre polynomials, represent the Mie angular functions. We calculated the angular scattered intensity using the Mie scattering amplitude functions for a LDPE particle with $r = 180$ μm at frequencies in the range 0.2 - 1 THz. Figure 1(a) illustrates the angular scattering patterns at frequencies $f = 0.2$, 0.4, 0.6, 0.8, and 1 THz, corresponding to size parameters $x = 0.6$, 1.3, 1.9, 2.5, and 3.1, respectively, which are all located in the Mie regime. It can be noted that the lowest frequency, 0.2 THz, yields the highest amounts of backscattering and scattering at oblique angles $\theta \neq 0°$, while as frequency increases, most of the radiation is scattered in the forward direction. This corroborates our measurements, where at higher detection angles, we could only extract resonances located at the lower frequencies. Figure 1(b) illustrates the scattered intensity $\frac{1}{2}(|S_1(\theta)|^2 + |S_2(\theta)|^2)$ between $\theta = -90°$ to $90°$, demonstrating that a particle-size scattering scenario results in different amounts of scattered intensity at different frequency components present in a broadband THz wave.

At high concentrations of scatterer particles ($> 10\%$), multiple scattering becomes the dominant process. In other words, the incident field on each particle represents a random superposition of the primary incident wave and the waves scattered by other particles in the medium. Effective medium theories incorporating the Mie formalism have been developed to predict the higher-order scattering contributions[3–5]. Here, we demonstrate the implementation of a self-consistent effective medium theory[4–7] for describing the frequency-dependent scattering loss by non-absorbent granular particles. Using the dynamic effective medium theory of Stroud and Pan[7], an effective dielectric constant ε_{eff} of a two-component composite material can be derived using an iterative scheme given by,

$$\varepsilon_{eff} = \varepsilon_1 \frac{A(\varepsilon_{eff})(1-V) + B(\varepsilon_{eff})}{A(\varepsilon_{eff}(1-V) - 2B(\varepsilon_{eff})}, \tag{3}$$

where

$$A(\varepsilon_{eff}) = i\frac{12\pi^2}{\lambda^2}\varepsilon_{eff}^{3/2}, \tag{4}$$

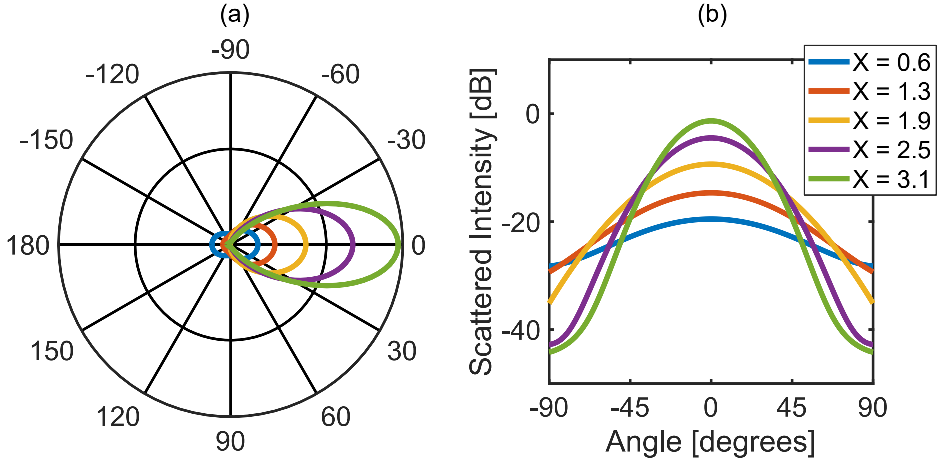

Figure 1. (a) A polar plot representing the angular scattering patterns generated by a LDPE particle with $r = 180\ \mu m$ at frequencies $f = 0.2$, 0.4, 0.6, 0.8, and 1 THz, corresponding to size parameters $x = 0.6$, 1.3, 1.9, 2.5,and 3.1, respectively, which are all located in the Mie regime. (b) The scattered intensity, $\frac{1}{2}(|S_1(\theta)|^2 + |S_2(\theta)|^2)$, for the LDPE particle at different size parameter values, demonstrating that the same particle generates different amounts of scattered intensity at different frequency components present in a broadband THz wave.

and

$$B(\varepsilon_{eff}) = \int \sum_n (2n+1)[a_n(r, \varepsilon_2/\varepsilon_{eff}) + b_n(r, \varepsilon_2/\varepsilon_{eff})]\rho(r)dr, \tag{5}$$

with V representing the volume fraction of the scatterers in the medium, λ representing the incident beam's wavelength, ε_1 representing the scatterers' dielectric constant, ε_2 representing the medium's dielectric constant, n is the number of Mie partial waves, a_n and b_n are the complex coefficients of Riccati-Bessel functions, and $\rho(r)$ represents the scatterer's size distribution. By assuming a fixed particle size, the size distribution can be written as,

$$\rho(r) = N\delta(r), \tag{6}$$

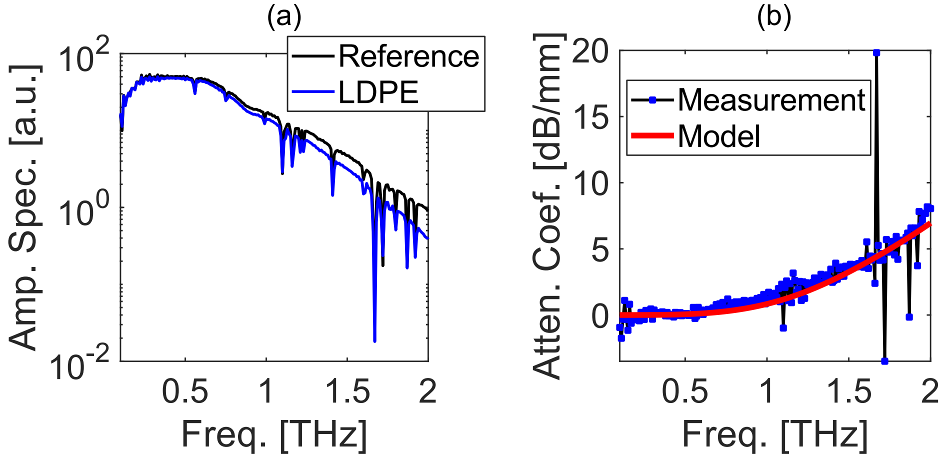

Figure 2. (a) The amplitude spectrum of a reference measurement through ambient air (solid black line) along with a measurement through 10 gr of loosely-packed LDPE powders (solid blue line) with mean particle radius $r = 25\ \mu m$ and volume density $V = 0.47$, (b) LDPE attenuation coefficient measured from the data shown in Fig. 2(a) (solid black line with squared blue markers) along the attenuation calculated from the Stroud and Pan's effective medium model, shown by the red line. Because LDPE is non-absorbent at THz frequencies, this attenuation in the pulse can be attributed to scattering. It is also evident that the model accurately predicts the scattering-induced attenuation in the measurement.

where $\delta(r)$ indicates the Kronecker delta function. The first approximation for this iterative implementation is obtained from the ordinary Bruggeman mixing rule given by,

$$(1-V)\frac{\varepsilon_1-\varepsilon_{eff}}{\varepsilon_1+2\varepsilon_{eff}}+V\frac{\varepsilon_2-\varepsilon_{eff}}{\varepsilon_2+2\varepsilon_{eff}}=0. \quad (7)$$

As an example, Fig. 2 illustrates the attenuation coefficient measured from loosely-packed LDPE powders with $r = 25\ \mu$m and volume density $V = 0.47$. Figure 2(a) illustrates the spectrum of a signal measured through ambient air, the reference measurement, and a signal measured through the LDPE powders. Figure 2(b) compares the measured attenuation coefficient, shown by the solid balck line with squared blue markers, with the attenuation coefficient obtained from the Stroud and Pan's dynamic effective medium model, shown by the solid red line. Because LDPE is non-absorbent at THz frequencies, the pulse attenuation can be attributed to scattering. It is also evident that the red line derived from the model can precisely predict the scattering-induced loss in the measurement.

For the more complicated scenario of having a pressed pellet with smaller particle size and larger scatterer density hidden beneath a turbid medium (0.3 volume fraction) with larger particle size, i.e., the test scenario studied in our manuscript, the effects of scattering by different media are convoluted. However, the discussed effective medium model can still predict the scattering-induced attenuation originated from the two media. Figure 3 shows the attenuation coefficients measured from four example HDPE pixels in the sample. Along each measured attenuation coefficient, shown by the blue lines, the red solid line demonstrates the attenuation coefficient derived from the effective medium model. It can be noted that the model accurately predicts the aggregate scattering attenuation caused by the two media.

1.2 Particle size

The particle size and scatterer density are not uniform across the sample. Hence, the mean value might not be representative of the correct particle size at different locations. However, Stroud and Pan's effective medium model can still be used to find

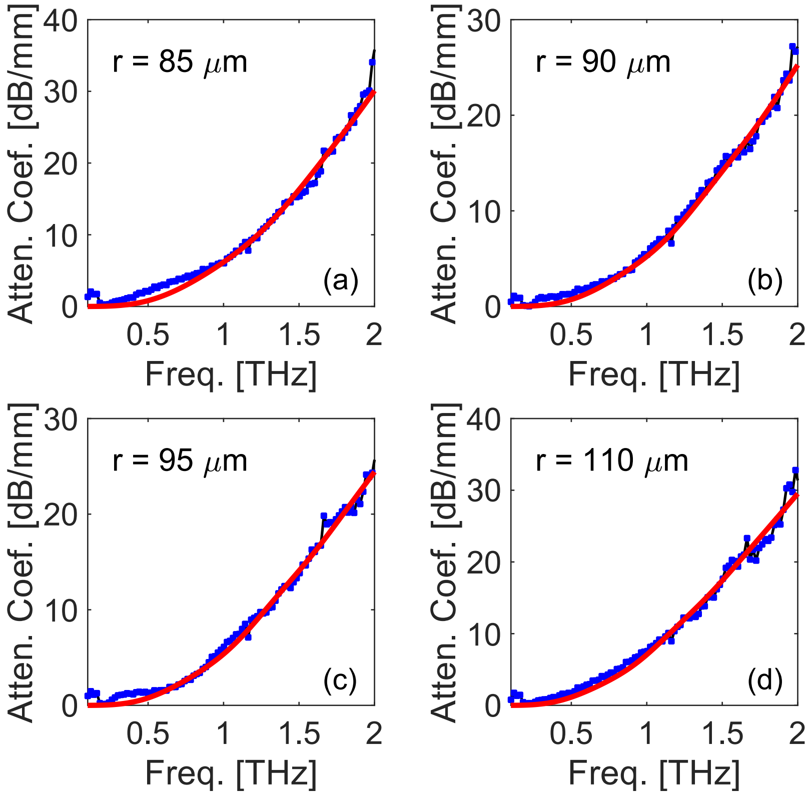

Figure 3. (a-d) Blue lines illustrate the attenuation coefficients measured through the HDPE pellet and LDPE particles from four random pixels selected from HDPE region of the sample, along with the attenuation coefficients calculated using the Stroud and Pan's effective medium model, shown by the red lines. Because both HDPE and LDPE are non-absorbent at THz frequencies, this high attenuation can be attributed to the Mie scattering. The average particle radius that yielded the minimum mean squared error between the measurement- and model-derived attenuation in each pixel is given in (a-d).

a more accurate particle size in each pixel by minimizing the mean squared error between the model and the measurement. Figure 3 demonstrates four examples of such pixels with the particle size derived from the model. It can be noticed that the radius (i.e., particle size/2) at each pixel is slightly different. Additionally, Fig. 4 illustrates the size parameter $x = 2\pi nr/\lambda$, which governs the scattering regime (x between 0.2 and 20 indicates the Mie regime[2]), for the size values obtained from the effective medium theory in Fig. 3. It confirms that across the measurements' useful bandwidth of 0.1 - 2 THz, these particles scatter according to the Mie theory.

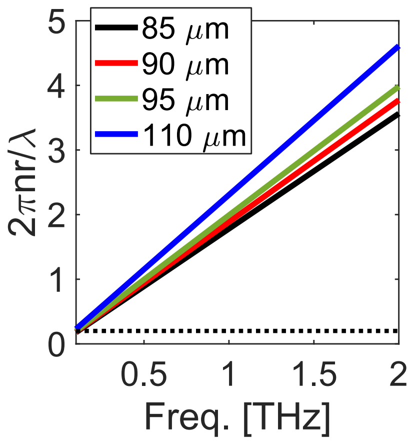

Figure 4. The size parameter $x = 2\pi nr/\lambda$ over frequencies 0.1 – 2 THz for the four particle radius values obtained from the Stroud and Pan's effective medium model in Fig. 3. The x values in the range 0.2 – 20 indicate the Mie regime. The horizontal dashed line delineates $x = 0.2$, confirming that the scattering regime remains in the Mie boundaries over our measurements' useful bandwidth.

2 Impact of the spectral properties of the resonant modes and noise on diffuse THz spectroscopy

We have shown that using the thresholded local maxima in the bimodality spectrum of the THz spectral images, the signature resonant frequencies of all the sample constituent chemicals can be extracted. Moreover, by applying this technique to the MODWT MRA of the THz extinction spectra, we recovered the resonant frequencies of the α-lactose monohydrate and PABA from diffuse THz waves at very low signal to noise ratios (SNR). Here, we further investigate the robustness of this technique in identifying the characteristic resonant frequencies from two overlapping resonant signatures. We also numerically explore the effects of the dielectric resonance properties, including the absorption mode's height and full width at half maximum (FWHM), on the performance of the bimodality-based diffuse spectroscopy technique. To study these effects, we used the α-lactose's dielectric function in the range 0.2 - 1.7 THz shown in Fig. 5(a). This dielectric function was acquired from a sample pellet purely made from α-lactose monohydrate. Additionally, using the Lorentz oscillator model of the classical dispersion theory, we created artificial resonant signatures in the proximity of the α-lactose's resonance at 0.53 THz.

The Lorentz oscillator model describes a medium as a compound of atomic dipole oscillators[8,9], and thereby the absorption lines in the dielectric function represent the uncoupled resonant modes of these oscillators. Accordingly, the dielectric function of a material with K resonant modes is given by

$$\varepsilon_r(f) = \varepsilon_\infty + \sum_{k=1}^{K} \frac{\Delta\varepsilon_k f_k^2}{f_k^2 - f^2 - i\frac{\gamma_k}{2\pi} f}, , \tag{8}$$

where ε_∞ is the dielectric constant at the higher-frequency limit of the bandwidth, and f_k is the kth resonant frequency, while $\Delta\varepsilon_k$ and γ_k represent the height and FWHM of the resonant signature at frequency f_k. The solid lines in Fig. 5(a) illustrate the Lorentz model fitted to the α-lactose's dielectric function using a 10-element parameter vector given by

$$p = \{\varepsilon_\infty, f_1, f_2, f_3, \Delta\varepsilon_1, \Delta\varepsilon_2, \Delta\varepsilon_3, \gamma_1, \gamma_2, \gamma_3\}, \tag{9}$$

where the subscripts 1, 2, and 3 correspond to the resonant modes at 0.53, 1.2, and 1.38 THz, respectively. Therefore, by excluding the parameters associated with the resonant modes at 1.2 and 1.38 THz from the Lorentz model, only the 0.53 THz resonant feature remains in the dielectric function. Moreover, by including new parameters in the Lorentz model, artificial

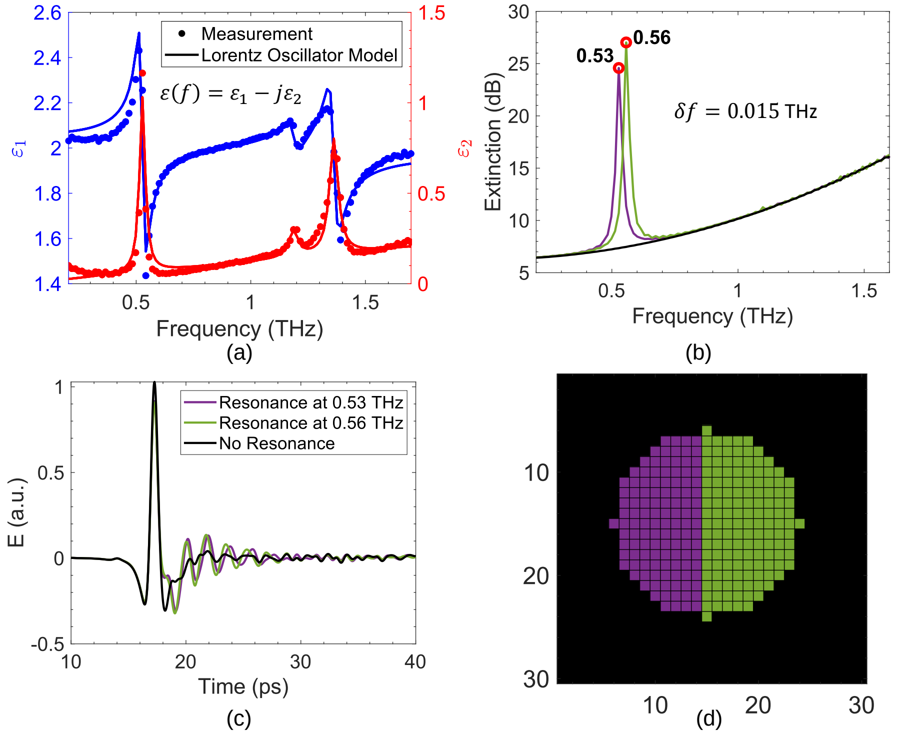

Figure 5. Creating resonant modes with desired properties, (a) The α-lactose monohydrate's dielectric function, $\varepsilon(f) = \varepsilon_r(f) - i\varepsilon_i(f)$, in the range 0.2 - 1.7 THz, while the Lorentz oscillator model, shown by the solid lines, is fitted to the measurements, (b) extinction spectra created from the α-lactose's resonance at $f_k = 0.53$ THz and shifting its center frequency to $f_k = 0.56$ THz in the Lorentz model, (c) time-domain THz signals corresponding to the extinction spectra shown in (b), (d) a binary sample hypothetically made from materials with a resonant frequency at 0.53 THz, purple part, and 0.56 THz, green part, while the surrounding material does have not any resonant frequencies, black part.

resonant signatures with desired properties can be created. For example, Fig. 5(b) illustrates two extinction spectra resultant from including only the 0.53 THz resonant signature and shifting its center position to 0.53+2δf THz in the α-lactose's Lorentz model, where $\delta f = 0.015$ THz represents the sampling period of the $\varepsilon(f)$. Therefore, we have simulated the dielectric function of an artifical material having a vibrational modes at 0.56 THz with variable resonance height and FWHM. Next, we simulate the THz extinction spectra of a 2 mm-thick sample comprised of both lactose and the new artificially modeled material in the presence of various noise scenarios. Fig. 5(c) shows the simulated THz time-domain signals corresponding to the extinction spectra of the two materials depicted in the sample, illustrated in Fig. 5(d). The left half of the sample is composed of the material with a resonant frequency at 0.53 THz, while its right half is made from the material with a resonance at 0.56 THz, and the surroundings are made from a material similar to polyethylene with no resonant frequencies, corresponding to the black lines in Figs. 5(b-c). Furthermore, using additive colored-noise models, we recreated wavelength-dependent low SNR scenarios often encountered in diffuse spectroscopy measurements. The color of a random noise model depends on the characteristics of its power spectral density (PSD), which is proportional to $1/f^{\beta}$ per unit of the bandwidth, where $\beta = 0$ produces white noise and $\beta = 1$ yields a pink noise model[10]. Because the scattering effects depend on the wavelength of the illuminating light, using a colored noise model with a frequency-dependent PSD, such as pink noise, better represents the experimental conditions described in this paper. Figures 6(a) and 6(c) compare the time-domain THz signals contaminated with additive pink noise at SNR = 10 dB and SNR = 0 dB, respectively. Note that although the model of the noise remains the same, the added noise is random and different for each pixel. Figures 6(b) and 6(d) illustrate the extinction spectra corresponding to these time-domain signals. It can be seen that the identification of the resonant features at 0.53 and 0.56 THz among other spectral artifacts is challenging, especially at SNR = 0 dB. At SNR = 0 dB, the noise contribution is dominant, which results in obscured resonant signatures. For example, the resonant feature at 0.56 THz is obscured in the green trace in Fig. 6(d). This corresponds with the measurements taken at the higher detection angles discussed in the main manuscript. Figures 6(e-f) illustrate that the proposed broadband bimodality spectrum of the sample shown in Fig. 5(d), when it is composed of the noise-contaminated THz signals, can still reveal the location of the resonant frequencies, even at a very low SNR. For example, Fig. 6(e) compares the bimodality

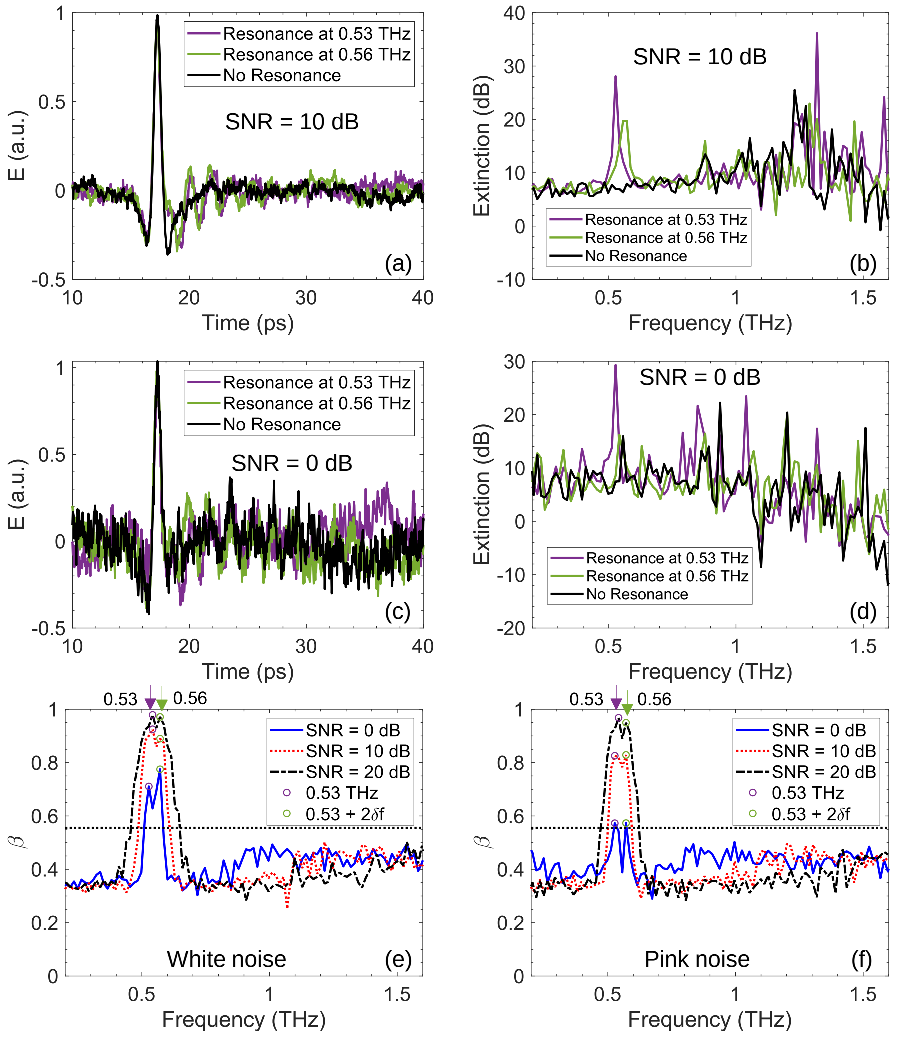

Figure 6. Bimodality spectrum in the presence of additive colored noise, (a), (c) the time-domain THz signals, shown in Fig. 5(c), contaminated with additive pink noise at SNR = 10 dB and SNR = 0 dB, (b), (d) extinction spectra corresponding to the signals shown in (a) and (c), (e) the bimodality spectrum of the sample shown in Fig. 5(d) when the time-domain signals are corrupted with white noise at SNR = 0, 10, and 20 dB, (f) the bimodality spectrum of the when signals are corrupted with pink noise at SNR = 0, 10, and 20 dB.

spectrum when the time-domain signals are corrupted with white noise at SNR = 0, 10, and 20 dB. In all three cases, the local maxima above the 5/9 threshold correspond to the resonant frequencies at 0.53 and 0.56 THz, while the bimodality at all other frequencies is below 5/9, even at SNR = 0 dB. Moreover, with decreasing SNR the bimodality at resonant frequencies also decreases, which is in accordance with the results obtained in the oblique measurement geometries in the main manuscript. Additionally, Fig. 6(f) illustrate the bimodality spectrum when signals are contaminated with additive pink noise at different SNRs. Although at SNR = 0 dB the local maxima at resonant frequencies are very close to the 5/9 threshold, they are still capable of revealing the resonant frequencies. Therefore, results in Fig. 6(e-f), considering the destructive noise effects on resonant signatures at lower SNRs shown in Figs. 6(b), confirm the robustness of the bimodality-based material detection.

Here, we address the problem of having more than two materials in the imaging target. We show the results obtained for the identification of three substances with different resonant frequencies. The resonances are simulated using the α-lactose monohydrate's Lorentz oscillator parameters at 0.53 THz. The first substance has a resonant frequency at 0.53 THz, similar to α-lactose, as shown in the dielectric function in Fig. 7(a). The dielectric function of the second and third substances are formed by shifting the resonant frequency to 0.6 and 0.75 THz, while the height and width of the resonant features (i.e., $\Delta\varepsilon_k$ and γ_k in Eq. (8)) are changed. Figure 7(b-c) show the refractive indices of the two new substances. Figure 7(d) shows an artificial sample made from these three substances, where the circle represents a pellet embedded in the background material.

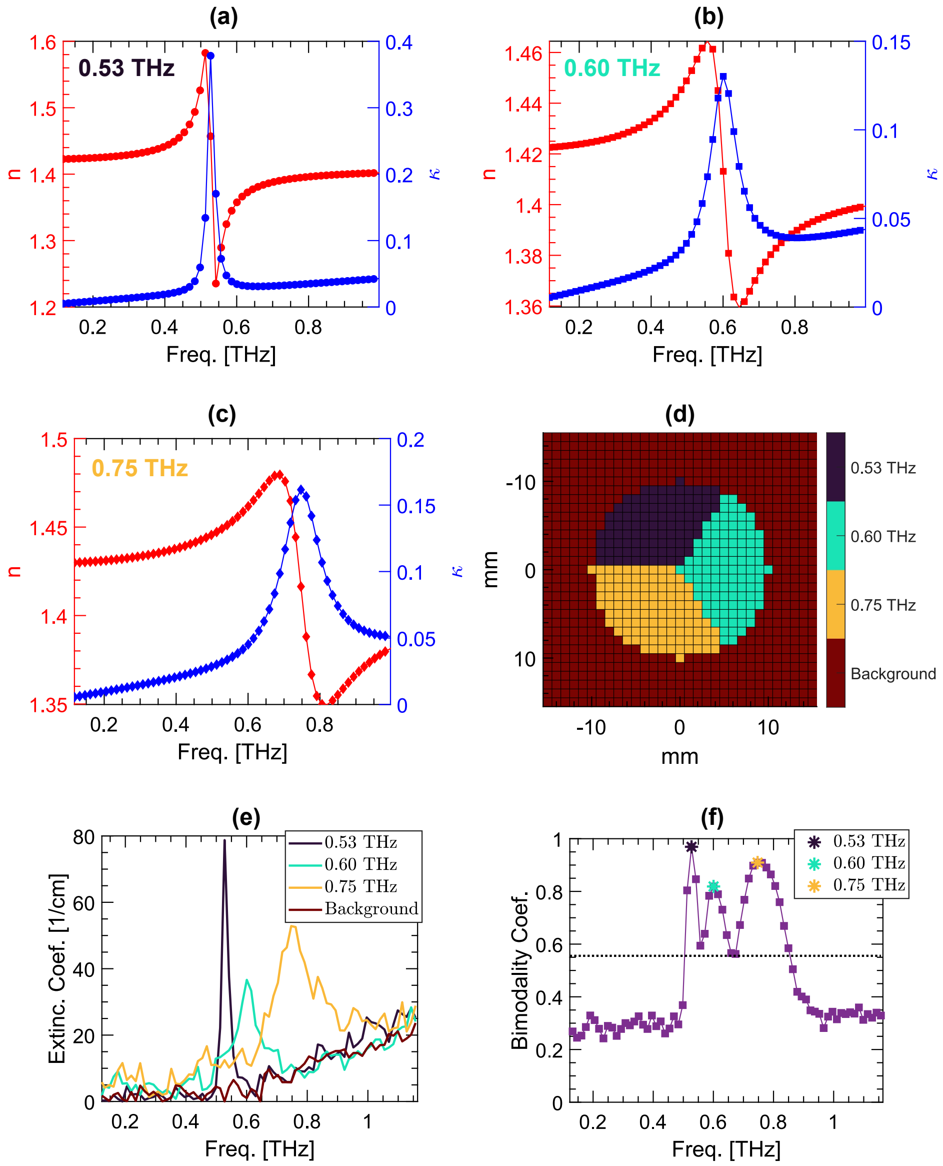

Figure 7. Identification of multiple substances using the bimodality coefficient spectrum, (a) the complex refractive index ($\tilde{n} = n - i\kappa$), of a material with a resonant frequency at 0.53 THz, (b) 0.6 THz, (c) 0.75 THz, the refractive indices are simulated using the Lorentz oscillator parameters of α-lactose monohydrate at 0.53 THz, while the height and width of the resonant features (i.e., $\Delta\varepsilon_k$ and γ_k in Eq. (8)) are changed, (d) an artificial sample pellet made from substances in (a-c), the black, cyan, and orange pixels designate the resonances at 0.53, 0.6, and 0.75 THz, whereas the background red pixels do not contain any resonances, (e) an example extinction coefficient from each region of the modeled sample, random pink noise at SNR = 20 dB is added to the extinction coefficients to replicate the frequency-dependent scattering artifacts, (f) the bimodality coefficient spectrum of the sample, where the frequency of the three local maxima above the 5/9 threshold, marked using black, cyan, and orange asterisks, correspond to the resonant frequencies at 0.53, 0.6, and 0.75 THz.

The black, cyan, and orange pixels designate the resonances at 0.53, 0.6, and 0.75 THz, respectively. The background red pixels do not exhibit any spectral resonances. We also added random pink noise to the extinction coefficients to artificially replicate the frequency-dependent scattering artifacts. Figure 7(e) shows an example extinction coefficient from each region. Figure 7(f) shows the bimodality coefficient spectrum of this sample. It can be noted that the frequency of the three local maxima above the 5/9 threshold, marked using black, cyan, and orange asterisks, correspond to the three simulated resonant frequencies.

Finally, we further investigate the effect of the height, $\Delta\varepsilon_k$, and FWHM, γ_k, of a resonant mode on the bimodality spectrum. With this aim, we varied the $\Delta\varepsilon_k$ and γ_k of the resonant signature at 0.56 THz from half to two times the value of the same

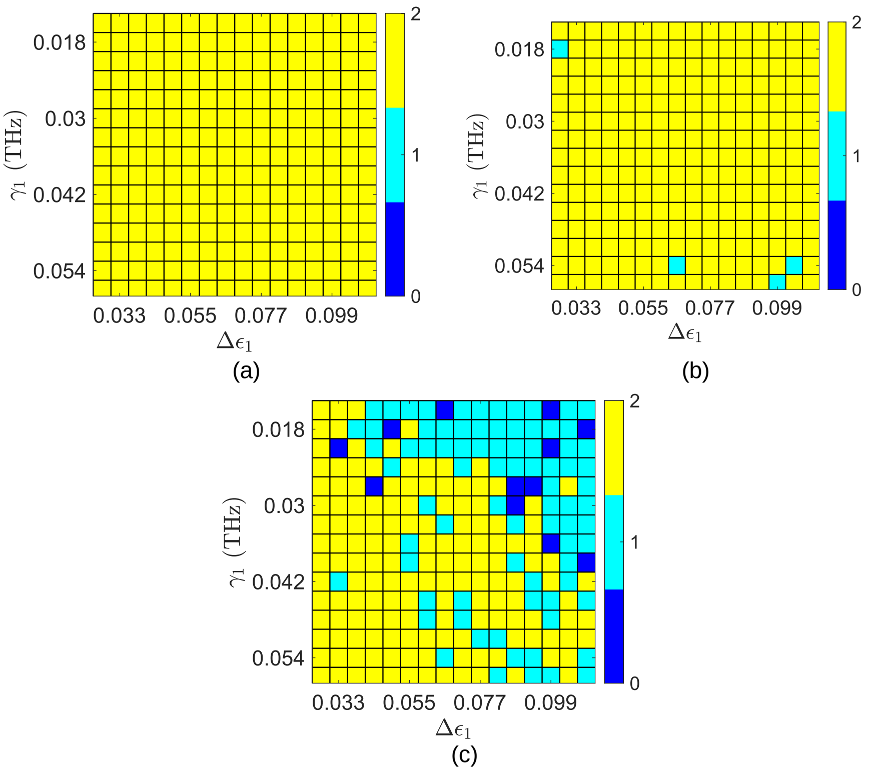

Figure 8. Bimodality-based resonance recognition dependence on the shape of the resonant modes, number of distinct resonant frequencies identified using the bimodality spectrum from two overlapping resonant modes with center frequencies at 0.53 and 0.56 THz, while the shape of the resonant mode at 0.56 THz, in the form of the resonant mode's height, $\Delta\varepsilon_k$, and FWHM, γ_k, changes, and the time-domain signals are contaminated with pink noise at (a) SNR = 20 dB, (b) SNR = 10 dB, and (c) SNR = 0 dB.

parameters for the α-lactose's resonant feature at 0.53 THz. Additionally, while changing the shape of the resonant mode, we contaminated the signals with pink noise at different SNRs and investigated the ability of the bimodality spectrum in differentiating the two resonant frequencies located at 0.53 and 0.56 THz. Figures 8(a-c) demonstrate the number of distinct resonant frequencies identified at each SNR for all combinations of the resonant mode's $\Delta\varepsilon_k$ and γ_k. Fig. 8(a) shows that at the SNR = 20 dB rate, for all the combinations of the height and FWHM values, the bimodality coefficient spectrum reveals two distinct resonant frequencies at 0.53 and 0.56 THz. However, as the noise SNR decreases in Figs. 8(b-c), the number of unidentified resonances increases, with the SNR = 0 dB showing the least number of correctly identified resonances. The color bars in Fig. 8 indicate the number of distinct vibrational modes identified in the overlapping bimodality spectra under each simulation scenario. These results indicate that the success of the proposed technique can be limited by the spectral proximity and overlap of the two very close resonant modes. However, at reasonable experimental noise levels (better than SNR = 0 dB) the new computational approach almost always identified two distinct but overlapping vibrational modes.

References

1. Bandyopadhyay, A. *et al.* Effects of scattering on thz spectra of granular solids. *Int. J. Inf. Milli. Waves* **28**, 969–978 (2007).
2. Ishimaru, A. *Wave propagation and scattering in random media*, vol. 2 (Academic press New York, 1978).
3. Zurk, L. M. *et al.* Terahertz scattering from granular material. *J. Opt. Soc. Am. B* **24**, 2238–2243 (2007).
4. Kaushik, M., Ng, B. W.-H., Fischer, B. M. & Abbott, D. Terahertz scattering by granular composite materials: An effective medium theory. *Appl. Phys. Lett.* **100**, 011107 (2012).
5. Kaushik, M., Ng, B. W.-H., Fischer, B. M. & Abbott, D. Terahertz scattering by dense media. *Appl. Phys. Lett.* **100**, 241110 (2012).

6. Chylek, P., Srivastava, V., Pinnick, R. G. & Wang, R. T. Scattering of electromagnetic waves by composite spherical particles: experiment and effective medium approximations. *Appl. Opt.* **27**, 2396–2404 (1988).

7. Stroud, D. & Pan, F. P. Self-consistent approach to electromagnetic wave propagation in composite media: Application to model granular metals. *Phys. Rev. B* **17**, 1602 (1978).

8. Born, M. & Wolf, E. *Principles of Optics: Electromagnetic Theory of Propagation, Interference and Diffraction of light* (Elsevier, 2013).

9. Kniffin, G. P. & Zurk, L. M. Model-based material parameter estimation for terahertz reflection spectroscopy. *IEEE Trans. Terahertz Sci. Technol.* **2**, 231–241 (2012).

10. Bendat, J. S. & Piersol, A. G. *Random Data: Analysis and Measurement Procedures*, vol. 729 (John Wiley & Sons, 2011).
